# Performance Comparison of Machine Learning Approaches on Hepatitis C Prediction Employing Data Mining Techniques

**DOI:** 10.3390/bioengineering10040481

**Published:** 2023-04-17

**Authors:** Azadeh Alizargar, Yang-Lang Chang, Tan-Hsu Tan

**Affiliations:** Department of Electrical Engineering, College of Electrical Engineering and Computer Science, National Taipei University of Technology, Taipei 10608, Taiwan; azadeh.tw@gmail.com (A.A.); ylchang@mail.ntut.edu.tw (Y.-L.C.)

**Keywords:** machine learning techniques, hepatitis C virus, data mining, decision tree, HCV, performance measurements, XGBoost, AUC

## Abstract

Hepatitis C is a liver infection caused by the hepatitis C virus (HCV). Due to the late onset of symptoms, early diagnosis is difficult in this disease. Efficient prediction can save patients before permeant liver damage. The main objective of this study is to employ various machine learning techniques to predict this disease based on common and affordable blood test data to diagnose and treat patients in the early stages. In this study, six machine learning algorithms (Support Vector Machine (SVM), K-nearest Neighbors (KNN), Logistic Regression, decision tree, extreme gradient boosting (XGBoost), artificial neural networks (ANN)) were utilized on two datasets. The performances of these techniques were compared in terms of confusion matrix, precision, recall, F1 score, accuracy, receiver operating characteristics (ROC), and the area under the curve (AUC) to identify a method that is appropriate for predicting this disease. The analysis, on NHANES and UCI datasets, revealed that SVM and XGBoost (with the highest accuracy and AUC among the test models, >80%) can be effective tools for medical professionals using routine and affordable blood test data to predict hepatitis C.

## 1. Introduction

There are many diseases affecting the human liver. One of the most important liver diseases is hepatitis C. It is caused by a virus that can be fatal if undetected. It can advance slowly and even cause cancer. In some cases, it can remain dormant in the body for even 10–20 years [1,2]. Some people with hepatitis C may only experience a brief illness, but for more than half of those who catch the virus, it progresses to become a chronic, life-long infection. Only 30% of hepatitis C virus infection patients recover on their own within six months, while most patients develop a chronic viral infection [3]. Two significant, potentially fatal health problems that can result from chronic hepatitis C are cirrhosis and liver cancer. Frequently, those who have chronic hepatitis C do not feel sick or exhibit any symptoms, and symptoms appear only when the disease is advancing. Patients may experience weakness, drowsiness, and dizziness, all of which might be mistaken for exhaustion brought on by work or study [4]. It is more dreadful when we find out that a hepatitis C vaccine is not yet available [5]. An antibody test is used to test individuals for HCV. Those who are positive for the antibody test should undergo a nucleic acid test (NAT) to find the HCV RNA for confirmation [6]. These two tests are effective in identifying and diagnosing patients with an active HCV infection. Most clinical laboratories can perform the HCV antibody test, which is reasonably priced and has a rapid turnaround time (TAT), but many patients who test positive for HCV antibody are lost to follow-up and never receive a NAT to confirm their diagnosis. A major barrier in the continuum of treatment is the two-step diagnostic process, which is burdensome for patients and results in a loss of patients due to follow-up. In addition, NAT, which is the gold standard for the diagnosis of HCV, is expensive and time-consuming. NAT is carried out by highly qualified personnel in special laboratories. Estimates show that around 84% of people do not have access to these laboratories. Even for those who do have access to these laboratories, the test itself is too expensive. These restrictions have a noticeable impact on low-income, resource-constrained environments [7].

WHO stated that medicines can cure around 95% of persons with hepatitis C infection, but access to diagnosis and treatment is way lower than this rate. If hepatitis C can be effectively predicted and treated in time, patients can survive. Sufficient Alanine aminotransferase (ALT), albumin (ALB), alkaline phosphatase (ALP), aspartate aminotransferase (AST), total bilirubin (TBIL), total protein (PROT), cholesterol (CHOL), serum cholinesterase (CHE), γ-glutamyl transferase (GGT), and creatinine blood (CREA) are the factors in the routine blood test used for diagnosing HCV by doctors. ALT is an enzyme that can indicate liver damage from liver diseases such as hepatitis, cirrhosis, infection, and liver cancer. A normal range for ALT is 4 to 36 U/L, and high levels may indicate liver disease. AST is another enzyme found mainly in the liver, with a normal range of 8 to 33 U/L. Increased AST levels in the bloodstream can signal liver cell damage. ALB measures the amount of albumin in the blood, with a normal range of 34 to 54 g/L. A low level of ALB in hepatitis C patients may indicate advanced liver disease. ALP is an enzyme that can indicate liver disease, with a normal range of 44 to 147 IU/L. However, doctors need to check other liver enzymes, since damage to other organs may also cause AST levels to rise. BIL measures the blood’s bilirubin amount and can increase when the liver becomes inflamed. PROT measures the amount of protein in the blood and can help identify liver and kidney diseases. CHOL can determine triglyceride and cholesterol levels in the blood, and having hepatitis C is associated with lower levels of LDL and cholesterol. CHE is an enzyme produced by hepatocytes, with its levels reflecting the liver’s synthetic activity. Finally, CREA measures how well the kidneys filter waste from the blood, which may indicate kidney damage associated with liver disease. The medical field is a data-rich area. Physicians may miss important information that is crucial for treatment and diagnosis of this disease. By using techniques based on machine learning which have been employed in different areas of the health sciences thus far, this problem can be solved. These techniques can be evaluated with big datasets. Different machine learning techniques, such as random forest (RF), support vector machines (SVM), artificial neural networks (ANN), linear regression, decision tree (DT), Logistic Regression, and K-nearest Neighbors (KNN) algorithms, have been utilized in various fields of medicine for the prediction of many diseases. Machine learning algorithms are becoming increasingly important in the field of healthcare, particularly in the prediction and prevention of hepatitis C infection. There are several reasons why using these algorithms to predict hepatitis C infection is important. Firstly, early detection and treatment of hepatitis C infection is critical for successful treatment and prevention of long-term complications. Machine learning models can help identify patients who are at high risk of infection and enable earlier interventions to prevent the spread of the disease. Secondly, accurately predicting hepatitis C infection can improve patient outcomes by enabling early diagnosis and treatment, reducing the risk of liver damage and other complications. Finally, machine learning models can help healthcare providers optimize resource allocation by identifying patients who are most likely to benefit from testing and treatment, reducing healthcare costs and improving patient outcomes. Overall, machine learning algorithms can play a vital role in predicting and preventing the spread of hepatitis C infection, improving patient outcomes, and optimizing healthcare resource allocation. 

Several studies have utilized machine learning algorithms to predict and diagnose hepatitis C. Ma et al. [8] established various classification models and found that the XGBoost algorithm had the best accuracy (91.56%). Ahammed et al. [9] implemented three machine learning algorithms and found that KNN had the best accuracy (94.40%). Nandipati et al. [10] found that binary class labels had better performance than multiclass labels in their study, and achieved an accuracy of 54.56% using the RF model. Mamdouh et al. [11] developed four machine learning models and found that the RF model had an accuracy of 94.06% without hyperparameter tuning and 94.88% with tuning. El-Salam et al. [12] used multiple-classifier models and achieved accuracy rates ranging from 65.6% to 68.9%. Hashem et al. [13] applied several machine learning approaches and found an accuracy range of 66.3% to 84.4% for predicting advanced chronic hepatitis C. Syafa’ah et al. [14] evaluated multiple algorithms and found that neural networks had the highest accuracy (95.12%). Oleiwi et al. [15] used four machine learning techniques and found that the decision tree method had the best accuracy (93.44%) for classifying and diagnosing hepatitis C. This study aims to choose the best algorithms for predicting hepatitis C based on routine and inexpensive blood test data.

The main contributions of this study are as follows:

- Using routine and inexpensive blood test data to predict hepatitis C.
- Using six machine learning algorithms (SVM, KNN, Logistic Regression, Decision Tree, XGBoost, ANN) to find appropriate models which can increase the prediction accuracy of hepatitis C.
- Replicating techniques on two different datasets to confirm the results.
- Calculating mean absolute error (MAE) in training and testing phases of six machine learning algorithms on two datasets to avoid overfitting.

## 2. Materials and Methods

**Figure 1:**
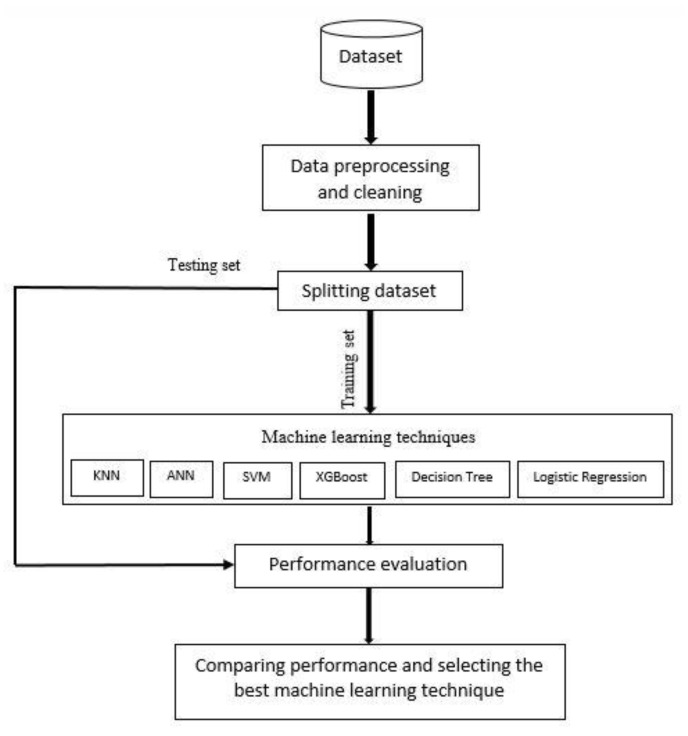
Proposed framework for hepatitis C prediction.

Three steps have been implemented in this study. In the first step, before using machine learning techniques, the description of the datasets, preprocessing, and feature importance are reviewed. The second step is to employ and develop various machine learning models. Finally in the last step, the performance of each model is evaluated in terms of confusion matrix, recall, precision, accuracy, F1 score, and the receiver operating characteristics (ROC), and the area under the curve (AUC), and the best model for the prediction of hepatitis C is selected after comparing the performance of these models. These models were developed with Python programs on Google Colab (Colaboratory). Figure 1 shows the proposed conceptual framework for the study.

### 2.1. Dataset Description

Two datasets were employed in this study. The NHANES dataset contains the data of 148 individuals and was obtained from the Centers for Disease Control (CDC) of the United States as a part of the National Health and Nutrition Examination Survey (NHANES) [16]. Ten features related to hepatitis C were selected from this dataset. These features are blood levels of ALT, ALB, ALP, AST, TBIL, PROT, CHOL, gender, age, and finally the presence of hepatitis C was confirmed by NAT.

The UCI dataset contains data from 615 individuals, was, created by Lichtinghagen et al. [17], and came from the Center for Machine Learning and Intelligent Systems at the University of California, Irvine (UCI). For each individual, a record that includes thirteen features, which are age, gender, blood levels of ALB, ALP, ALT, AST, bilirubin (BIL), CHE, CHOL, GGT, PROT, CREA, and the target feature categorizes individuals as either blood donors or those with hepatitis C, including its progression to fibrosis and cirrhosis. 

### 2.2. Data Preprocessing and Feature Importance

As data quality is a crucial consideration in the data mining process for disease prediction and diagnosis, a data cleaning procedure was used to make our datasets more accurate for the prediction. Some attributes in these two datasets had some missing and duplicate values. In this study, median imputation was used to fill in missing values in the datasets. This method was chosen because it is a simple and widely used technique for handling missing data, especially when the missing values are believed to be missing at random. The median is a robust measure of central tendency that is not affected by outliers and is less likely to introduce bias in the analysis than other methods such as mean imputation. Previous research has also shown that median imputation can perform well when compared to other imputation methods in terms of reducing bias and increasing the accuracy of the analysis [18,19]. Additionally, median imputation has been used in various medical studies for handling missing data. Therefore, based on the simplicity and robustness of the method, as well as its success in previous research, median imputation was deemed appropriate for this study.

Data standardization was applied to the datasets after dealing with missing data, deleting duplicates, and converting some string features to numeric. Feature-importance techniques, which are techniques that rate input characteristics according to how well they can predict a target variable, were the next step. In this study, the embedded methods were used to calculate the feature importance.

In machine learning, an embedded method is a feature-selection technique that selects the most relevant features during the model training process. This is achieved by incorporating feature selection into the algorithm used for training the model, rather than performing feature selection as a separate preprocessing step.

Embedded methods work by evaluating the importance of features during the model training process and selecting only those that contribute the most to the accuracy of the model. This is typically achieved by assigning a weight or coefficient to each feature based on its importance in the model [20].

### 2.3. Model Selection

In this study, we have employed various machine learning techniques to predict hepatitis C using common and affordable blood test data to diagnose and treat patients in the early stages. To achieve this goal, we have carefully selected six machine learning algorithms, including decision tree, ANN, KNN, Logistic Regression, SVM, and XGBoost, based on their proven efficacy in medical diagnosis [21,22]. For each algorithm, we have used the hyperparameters to ensure maximum accuracy. For instance, we have used C = 1.0, gamma = scale, kernel function = radial basis function (RBF) for SVM, and objective = binary: logistic, learning_rate = 0.2 for XGBoost. Similarly, we have employed the activation function = relu, hidden layer = 3, epochs = 100 for ANN, n_neighbors = 10 for KNN, splitter = best for decision tree, and penalty = l2, solver= lbfgs for Logistic Regression. The choice of these hyperparameters played a crucial role in achieving the high accuracy of our models, and this study could serve as a reference for future researchers in this field.

### 2.4. Evaluation of Model Performance

Various statistics were used for the evaluation of the developed machine learning algorithms. This information includes True Positives (TP), False Positives (FP), True Negatives (TN), and False Negatives (FP) with seven performance measurements, which are confusion matrix, accuracy, precision, F1 score, recall, the receiver operating characteristics (ROC), and the area under the curve (AUC). 

The probability that a disease exists when the result is positive is referred to as the positive predictive value, and when the outcome is negative, the probability that a disease does not exist is referred to as the negative predictive value. The confusion matrix is used to provide a summary of the prediction outcomes. 

The accuracy is determined by how close the obtained results are to the real value of the measurement. For this reason, accuracy can also be computed as follows:

accuracy = (TP + TN)/(TP + TN + FP + FN)

The precision measures the proportion of positive class predictions that really are in the positive class, which is calculated with the following equation:

Precision = TP/(TP + FP)

The recall is a metric used in machine learning to evaluate the ability of a model to correctly identify all positive instances in a dataset. Recall is defined mathematically as follows.

recall = TP/(TP + FN)

The F1 score is a metric to evaluate the balance between precision and recall of a model. It can be calculated as follows:

F1_score = TP/(TP + 1/2(FP + FN))

A ROC curve (receiver operating characteristic curve) indicates the trade-off between the true positive rate and the false positive rate where the true positive rate is used to calculate the proportion of true positives that are correctly identified, and the false positive rate is determined as the percentage of negative events incorrectly classified as positive to the total number of negative events. The area under the curve (AUC), which serves as a summary of the ROC curve, is a measurement of a classifier’s capacity to distinguish between classes. A higher AUC means a stronger ability to differentiate between positive and negative classifications.

## 3. Results

In total, 148 (59 female and 89 male) and 615 (238 female and 377 male) individuals were included in the NHANES and UCI datasets, respectively. The NHANES dataset and UCI dataset have ten and thirteen features, respectively. Table 1 illustrates the features of the data information and analysis.

**Figure 2:**
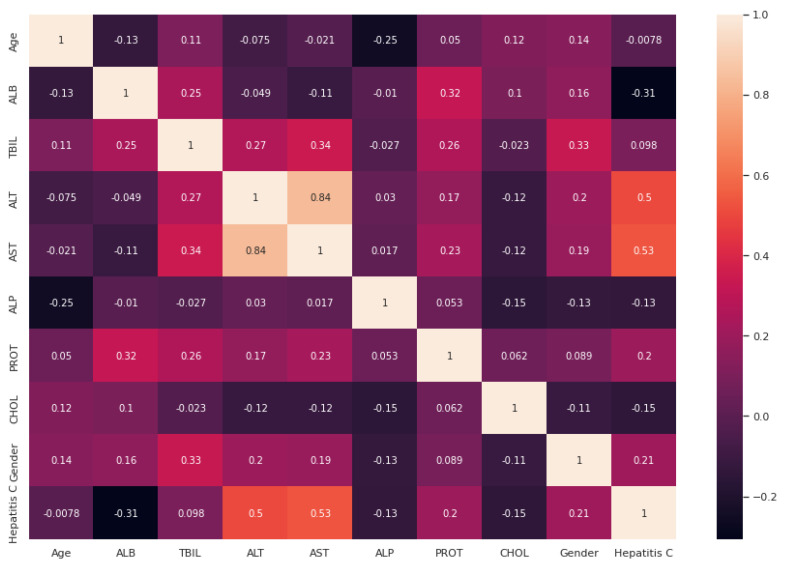
Heat map for the relation between different attributes based on the NHANES dataset.

**Figure 3:**
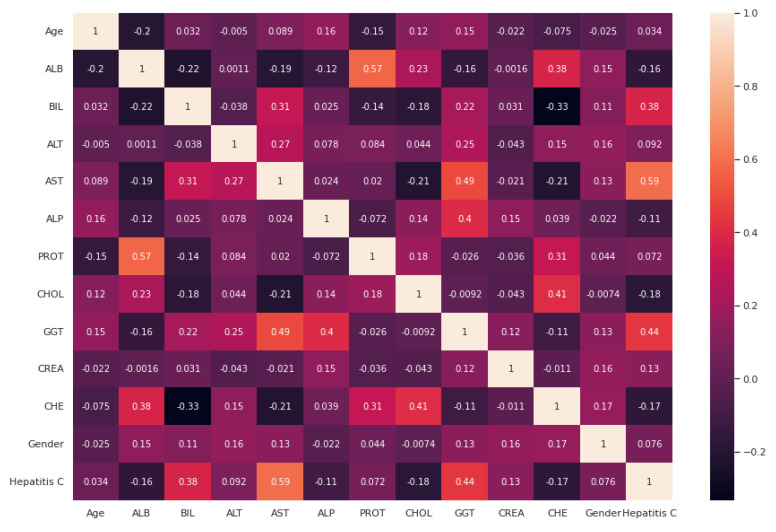
Heat map for the relation between different attributes based on the UCI dataset.

**Figure 4:**
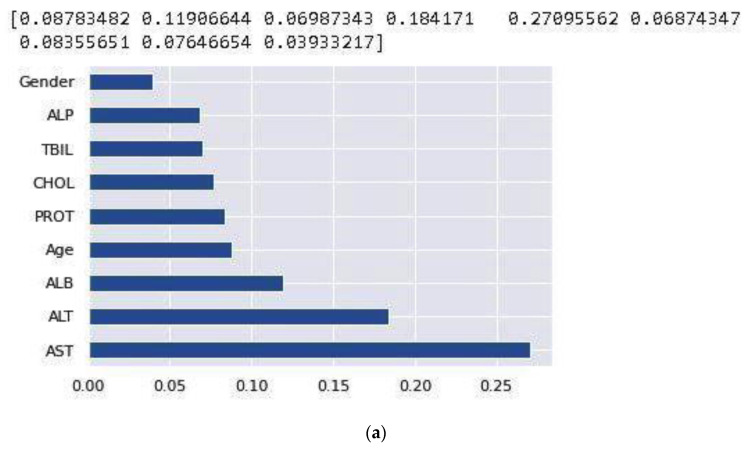

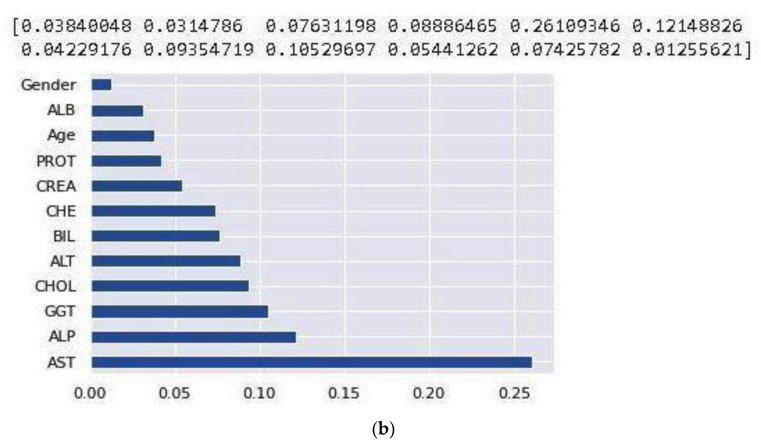
Feature importance based on the NHANES (**a**) and UCI (**b**) datasets.

After handling the missing data, removing the duplicates, and converting some string features to numeric, data standardization was applied to the datasets. Correlation analysis was performed afterwards. The heat maps illustrated in Figure 2 and Figure 3 are utilized in this work to show the correlation between the features in these two datasets. After that, the value of each feature importance was computed for ranking the features by embedded methods. The rank of features’ importance in the datasets are shown in Figure 4. According to this analysis, among these features, gender has the least importance and AST has the most importance in these two datasets. 

**Figure 5:**
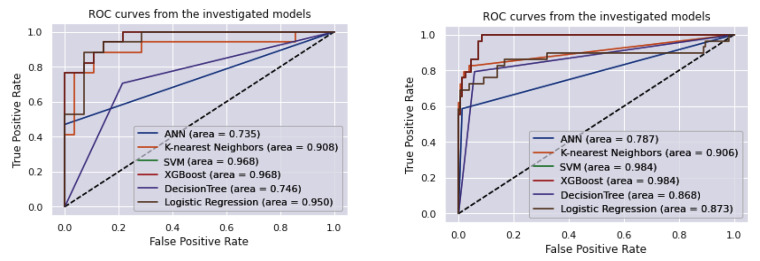
ROC curve and AUC obtained by six machine learning algorithms employing the NHANES (**left**) and UCI (**right**) datasets.

In this research, the datasets were, respectively, split into 30% and 70% for the testing set and training set. To find a technique that is appropriate to predict hepatitis C, the results of the six machine learning techniques are evaluated and compared. Table 2 and Table 3 show the comparative result of the algorithms using two datasets. Precision, recall, and F1 score are listed for all the labels (0 means no hepatitis C and 1 means hepatitis C). Figure 5 shows ROC and AUC obtained by these six machine learning algorithms employing the NHANES and UCI datasets. The comparison of the predicted values between the implemented techniques and the actual values is demonstrated in the confusion matrix. The models utilized in this study are evaluated using a confusion matrix (Table 4). The confusion matrix figures for these algorithms are included in the Supplementary Materials, with Figure S1 showing the NHANES dataset and Figure S2 showing the UCI dataset. Calculating the mean absolute error (MAE) for both training and testing datasets is a common practice in machine learning to avoid overfitting. Overfitting occurs when a model is too complex and fits the training data too closely, resulting in poor performance on new data. By calculating the MAE for both training and testing datasets, the performance of the model can be assessed and compared to determine if the model is overfitting to the training data [23]. The MAE is given in Table 5 for the both training and testing datasets.

## 4. Discussion

Based on the experimental results, SVM and XGBoost techniques can be used as efficient tools for doctors and specialists using routine and inexpensive blood test data to predict hepatitis C. Through the years, machine learning techniques have been used in disease prediction, such as with hepatitis C, by various researchers. L. Ma et al. [8] established various classification models on 615 individuals’ data to predict hepatitis C patients. In their study, the XGBoost algorithm outperforms other models with an accuracy of 91.56%. Our results also confirm that the XGBoost algorithm is an accurate model for predicting hepatitis C. Ahammed et al. [9] implemented three machine learning algorithms including RF, SVM, and KNN on a hepatitis C dataset, and KNN showed the best accuracy (94.40%) among all models. In research in 2020 using an Egyptian hepatitis C dataset, Nandipati et al. [10] found that binary class labels had better performance compared to multiclass labels. The highest accuracy in their study was 51.06% using a KNN model and 54.56% using an RF model in multi- and binary class labels. Our study is in accordance with this study.

H. Mamdouh et al. [11] developed four machine learning models including naive Bayes (NB), RF, KNN, and Logistic Regression to predict hepatitis C with a dataset of 859 patients. The RF model reached the accuracy of 94.06% without and 94.88% with adjusting for the hyperparameter values of the RF classifier. 

M. Abd El-Salam et al. [12] tried to develop an efficient technique for diagnosing esophageal varices disease, leading to timely patient treatment. The researchers employed well-known classifier models such as neural networks, naive Bayes, SVM, RF, and Bayesian networks (BN). They used 24 clinical laboratory variables of 4962 hepatitis C individuals in Egypt between 2006 and 2017. The accuracy rates employing the SVM, RF, C4.5, Multi-Layer Perceptron (MLP), NB, and BN algorithms were 67.8%, 66.3%, 67.2%, 65.6%, 66.7%, and 68.9%, respectively. Our results have better accuracy than this study.

S. Hashem et al. [13] applied several machine learning approaches to develop classification models for the prediction of chronic hepatitis C using clinical data. Decision trees, multilinear regression, particle swarm optimization, and genetic algorithms models were developed to predict advanced chronic hepatitis C. For evaluating how well the recommended models performed, the ROC was used. With an accuracy range from 66.3% to 84.4% and an AUROC of 0.73 to 0.76, these algorithms were able to successfully predict advanced chronic hepatitis C in patients with hepatocellular carcinoma (HCC), which is the most typical primary form of liver cancer. Our study is in accordance with this study. 

L. Syafa’ah et al. [14] evaluated the algorithms KNN, naive Bayes, neural networks, and RF for detection of hepatitis C. The results demonstrated that NN’s accuracy can reach 95.12%, higher than naive Bayes, KNN, and RF. Another study, by Oleiwi et al. [15], used four machine learning techniques (KNN, SVM, naive Bayes, and decision tree). They used these models for prediction, classification, and diagnosis of hepatitis C on 615 records. Results indicated that the decision tree method, with accuracy of 93.44%, performed better in terms of the given classification goals compared to other models. 

Although the heat map plots showed that age and gender have weak correlations with other features, there is some evidence and studies show that they are important factors related to hepatitis C. A systematic review and meta-analysis was conducted by Muhammad Abdel-Gawad et al. [24] to evaluate and analyze the gender differences in hepatitis C regarding the virus infection rates. They found that the HCV RNA positivity rate is significantly higher in male adults than female. Moreover, a study by Rachel Baden et al. [25] shows that infection with the hepatitis C virus affects men disproportionately more than women. Women also progress at a slower rate than men if they become chronically infected. 

Saputra et al. [26] used the random forest algorithm to detect hepatitis C and achieved an accuracy of 92.5%, while Li et al. [27] developed a hepatitis C virus detection model using random forest, Logistic Regression, and an ABC algorithm, and achieved a best accuracy of 94.5%. Terlapu et al. [28] developed an intelligent diagnosis system for hepatitis C using a probabilistic neural network-based approach, and achieved an accuracy of 96.4%. Kaunang et al. [29] compared the performance of various machine learning algorithms in predicting hepatitis C, including decision tree, random forest, naive Bayes, and SVM. The SVM algorithm demonstrated the highest accuracy of 94.5%. Safdari et al. [30] applied data mining techniques to classify patients with suspected hepatitis C virus infection and achieved an accuracy of 90.3%. Akter et al. [31] developed a machine learning model to detect the progression of hepatitis C virus in patients’ liver condition and achieved an accuracy of 91.67%. In comparison to the aforementioned six studies, our analysis on the NHANES and UCI datasets found that SVM and XGBoost (with AUC, >80%) can effectively predict hepatitis C using routine and affordable blood test data. To the existing literature, our work adds the development of more accurate and efficient diagnostic tools for predicting hepatitis C.

Age is a condition that leads to a gradual loss of the body’s ability to maintain homeostasis, making it a significant risk factor for chronic diseases. Studies have shown that hepatitis C can damage various organs, particularly the liver, and that this damage worsens with age. Compared to an older adult, a younger person may survive for decades with minimal or no liver damage. However, as individuals age, their immune system weakens, and the rate of fibrosis often accelerates [32]. Although the heat maps show low correlation with other variables, excluding these two variables might lead to bias in our study and including these two variables can be considered as one of the advantages in our study.

One of the strengths of this study is using the NHANES dataset that includes data from individuals of different races and ethnicities in the United States. NHANES is designed to be representative of the non-institutionalized civilian population in the U.S., and oversamples certain demographic groups to ensure adequate representation. Another advantage of this study is that two datasets were utilized to both train and test the algorithms, and the results were compared among various models. While the models’ accuracies were deemed satisfactory, it would be advantageous for other researchers to replicate the study’s techniques on different datasets to confirm the results.

One disadvantage of this study is that not all critical features could be retained in both datasets, which is typical of secondary data analysis.

## 5. Conclusions

In conclusion, our study aimed to employ various machine learning techniques to predict hepatitis C based on routine and affordable blood test data. Our results demonstrated that the SVM and XGBoost techniques were effective in diagnosing hepatitis C in its early stages with high accuracy and AUC (>80%). Our analysis also identified important features such as ALT, ALB, ALP, AST, TBIL, PROT, CHOL, CHE, GGT, CREA, gender, and age that could be used in these techniques. However, our study has some limitations, including the use of limited datasets, the lack of clinical data, and the absence of a clinical trial. In future work, we aim to incorporate additional features related to hepatitis C to develop more reliable and efficient machine learning techniques. Moreover, we recommend conducting a clinical trial to validate the performance of these techniques in real-world scenarios. Overall, our study provides promising results for the early detection and diagnosis of hepatitis C using machine learning techniques, which could ultimately improve patient outcomes and save lives.

## Figures and Tables

**Table 1:** Description of features of the datasets.

Features	NHANES Dataset			UCI Dataset		
	Mean	std	Range	Mean	std	Range
ALT (U/L)	29.37	26.86	3–189	28.42	25.45	0.90–325.3
ALB (g/L)	38.91	4.07	26–48	41.62	5.77	14.90–82.20
ALP (U/L)	91.84	40.77	42–363	67.40	26.19	1.90–416.60
AST (U/L)	30.79	23.33	6–134	34.78	33.09	10.60–324
TBIL (μmol/L)	7.90	4.53	1.71–29.07	_	_	_
PROT (g/L)	72.65	4.91	61–85	71.99	5.50	44.80–90.00
CHOL (U/L)	4.61	1.04	2.09–8.14	5.32	1.20	0.00–9.67
Age (years)	56.81	15.84	12–80	47.40	10.05	19–77
GGT (U/L)	_	_	_	39.53	54.66	4.50–650.95
BIL (μmol/L)	_	_	_	11.39	19.67	0.80–254
CREA (μmol/L)	_	_	_	81.28	49.75	8–1079.10
CHE (U/L)	_	_	_	8.19	2.20	1.42–16.41

ALB: albumin; ALP: Alkaline phosphatase; ALT: alanine aminotransferase; AST: aspartate aminotransferase; TBIL: total bilirubin; CHE: serum cholinesterase; PROT: total protein; CHOL: cholesterol; GGT: γ-glutamyl transferase; CREA: creatinine blood (CREA); BIL: bilirubin; std: Standard deviation.

**Table 2:** Comparative result of the six machine learning algorithms on the NHANES dataset.

Machine Learning Algorithms	Accuracy	Machine Learning Algorithm Result						Area under the Curve
		Precision		Recall		F1-Score		
		0	1	0	1	0	1	
K-nearest Neighbors classifier (KNN)	0.78	0.74	1.00	1.00	0.41	0.85	0.58	0.908
Decision Tree	0.76	0.81	0.67	0.79	0.71	0.80	0.69	0.746
Artificial Neural Network (ANN)	0.80	0.76	1.00	1.00	0.47	0.86	0.64	0.735
Support Vector Machine (SVM)	0.82	0.78	1.00	1.00	0.53	0.88	0.69	0.968
Logistic Regression	0.78	0.76	0.82	0.93	0.53	0.84	0.64	0.950
Extreme Gradient Boosting (XGBoost)	0.82	0.78	1.00	1.00	0.53	0.88	0.69	0.968

**Table 3:** Comparative result of the six machine learning algorithms on the UCI dataset.

Machine Learning Algorithms	Accuracy	Machine Learning Algorithm Result						Area under the Curve
		Precision		Recall		F1-Score		
		0	1	0	1	0	1	
K-nearest Neighbors classifier (KNN)	0.89	0.88	1.00	1.00	0.28	0.94	0.43	0.906
Decision Tree	0.92	0.96	0.72	0.94	0.79	0.95	0.75	0.868
Artificial Neural Network (ANN)	0.92	0.93	0.89	0.99	0.59	0.96	0.71	0.787
Support Vector Machine (SVM)	0.95	0.96	0.92	0.99	0.76	0.97	0.83	0.984
Logistic Regression	0.94	0.94	0.95	0.99	0.66	0.97	0.78	0.873
Extreme Gradient Boosting (XGBoost)	0.95	0.96	0.92	0.99	0.76	0.97	0.83	0.984

**Table 4:** Numbers of TP, FP, TN, and FN cases of the six machine learning algorithms on the two datasets (the figures of the confusion matrices of these algorithms are shown in the Supplementary Materials, (Figure S1) NHANES Dataset, (Figure S2) UCI dataset).

	NHANES Dataset				UCI Dataset			
Machine Learning Techniques	TP	FN	FP	TN	TP	FN	FP	TN
K-nearest Neighbors (KNN)	7	10	0	28	8	21	0	156
Logistic Regression	9	8	2	26	19	10	1	155
Support Vector Machine (SVM)	9	8	0	28	22	7	2	154
Artificial Neural Networks (ANN)	8	9	0	28	17	12	2	154
Extreme Gradient Boosting (XGBoost)	11	6	0	28	21	8	1	155
Decision Tree	12	5	6	22	23	6	9	147

**Table 5:** Mean absolute error (MAE) in training and testing phases of the six machine learning algorithms on the two datasets.

Machine Learning Techniques	Mean Absolute Error			
	NHANES Dataset		UCI Dataset	
	Training	Testing	Training	Testing
Logistic Regression	0.05	0.08	0.23	0.23
K-nearest Neighbors (KNN)	0.00	0.10	0.00	0.31
Support Vector Machine (SVM)	0.02	0.07	0.22	0.25
Artificial Neural Networks (ANN)	0.33	0.71	0.4	0.3
Extreme Gradient Boosting (XGBoost)	0.00	0.06	0.04	0.20
Decision Tree	0.00	0.07	0.00	0.26

## Data Availability

The NHANES dataset, the Centers for Disease Control (CDC) of the United States as a part of the National Health and Nutrition Examination Survey (NHANES), available online at https://wwwn.cdc.gov/nchs/nhanes/continuousnhanes/default.aspx?BeginYear=2017 (accessed on 15 February 2020). The UCI dataset, the Center for Machine Learning and Intelligent Systems at the University of California, Irvine (UCI) available online at https://archive.ics.uci.edu/ml/datasets/HCV+data (accessed on 15 February 2019).

## Supplementary Materials

The following supporting information can be downloaded at: https://www.mdpi.com/article/10.3390/bioengineering10040481/s1, Figure S1: Confusion matrix of six machine learning algorithms with the NHANES Dataset.; Figure S2: Confusion matrix of six machine learning algorithms with the UCI dataset.

## References

[1] Abrantes J., Torres D.S., Brandão-Mello C.E. (2020). The Many Difficulties and Subtleties in the Cognitive Assessment of Chronic Hepatitis C Infection. Int. J. Hepatol..

[2] Hepatitis C., Gerber M.A., World Health Organization (1994). Pathology of Hepatitis C. FEMS Microbiol. Rev..

[3] Zhao Z., Chu M., Guo Y., Yang S., Abudurusuli G., Frutos R., Chen T. (2022). Feasibility of Hepatitis C Elimination in China: From Epidemiology, Natural History, and Intervention Perspectives. Front. Microbiol..

[4] Modi A.A., Liang T.J. (2006). Hepatitis C: A Clinical Review. J. Med. Virol..

[5] Zingaretti C., De Francesco R., Abrignani S. (2014). Why is it so difficult to develop a hepatitis C virus preventive vaccine?. Clin. Microbiol. Infect..

[6] Centers for Disease Control and Prevention of the United States Web-Site (CDC) https://www.cdc.gov/hepatitis/hcv/index.htm.

[7] HCV Testing. https://www.healio.com/news/hepatology/20200702/hcv-testing-gold-standard-vs-hcv-core-antigen-testing.

[8] Ma L., Yang Y., Ge X., Wan Y., Sang X. Prediction of Disease Progression of Chronic Hepatitis C Based on XGBoost Algorithm. Proceedings of the 2020 International Conference on Robots & Intelligent System (ICRIS).

[9] Ahammed K., Satu M.S., Khan M.I., Whaiduzzaman M. Predicting Infectious State of Hepatitis C Virus Affected Patient’s Applying Machine Learning Methods. Proceedings of the 2020 IEEE Region 10 Symposium (TENSYMP).

[10] Nandipati S.C., XinYing C., Wah K.K. (2020). Hepatitis C Virus (HCV) Prediction by Machine Learning Techniques. Appl. Model. Simul..

[11] Mamdouh H., Shams M., Abd El-Hafeez T. (2022). Hepatitis C Virus Prediction Based on Machine Learning Framework: A Real-World Case Study in Egypt. Knowl. Inf. Syst..

[12] Abd El-Salam S.M., Ezz M.M., Hashem S., Elakel W., Salama R., ElMakhzangy H., ElHefnawi M. (2019). Performance of Machine Learning Approaches on Prediction of Esophageal Varices for Egyptian Chronic Hepatitis C Patients. Inform. Med. Unlocked.

[13] Hashem S., Esmat G., Elakel W., Habashy S., Raouf S.A., Elhefnawi M., Eladawy M.I., ElHefnawi M. (2018). Comparison of Machine Learning Approaches for Prediction of Advanced Liver Fibrosis in Chronic Hepatitis C Patients. IEEE/ACM Trans. Comput. Biol. Bioinform..

[14] Syafa’ah L., Zulfatman Z., Pakaya I., Lestandy M. (2021). Comparison of Machine Learning Classification Methods in Hepatitis C Virus. J. Online Inform..

[15] Shi L., Wei L., Tao Y., Oleiwi A. (2020). Development of Diagnostic Decision Making For Chronic Hepatitis C Virus Patients By Various Supervised Predictive Model. J. Adv. Res. Dyn. Control Syst..

[16] CDC Database. https://wwwn.cdc.gov/nchs/nhanes/continuousnhanes/default.aspx?BeginYear=2017.

[17] (2020). HCV Data Data Set. UCI Machine Learning Repository. https://archive.ics.uci.edu/ml/datasets/HCV+data.

[18] Schafer J.L., Graham J.W. (2002). Missing Data: Our View of the State of the Art. Psychol. Methods.

[19] Van Buuren S. (2012). Flexible Imputation of Missing Data.

[20] Li J., Zhang H., Zhao J., Guo X., Rihan W., Deng G. (2022). Embedded Feature Selection and Machine Learning Methods for Flash Flood Susceptibility-Mapping in the Mainstream Songhua River Basin, China. Remote Sens..

[21] Faris H., Aljarah I., Al-Madi N., Mirjalili S. (2016). Optimizing the Learning Process of Feedforward Neural Networks Using Lightning Search Algorithm. Int. J. Artif. Intell. Tools.

[22] Habib A.-Z.S.B., Tasnim T., Billah M.M. A Study on Coronary Disease Prediction Using Boosting-Based Ensemble Machine Learning Approaches. Proceedings of the 2019 2nd International Conference on Innovation in Engineering and Technology (ICIET).

[23] Montesinos López O.A., Montesinos López A., Crossa J., Montesinos López O.A., Montesinos López A., Crossa J. (2022). Overfitting, Model Tuning, and Evaluation of Prediction Performance BT—Multivariate Statistical Machine Learning Methods for Genomic Prediction.

[24] Abdel-Gawad M., Nour M., El-Raey F., Nagdy H., Almansoury Y., El-Kassas M. (2023). Gender Differences in Prevalence of Hepatitis C Virus Infection in Egypt: A Systematic Review and Meta-Analysis. Sci. Rep..

[25] Baden R., Rockstroh J.K., Buti M. (2014). Natural History and Management of Hepatitis C: Does Sex Play a Role?. J. Infect. Dis..

[26] Saputra T.A.N., Arizona K.I., Andrian M.R., Kurniadi F.I., Juarto B. Random Forest in Detecting Hepatitis C. Proceedings of the 2022 9th International Conference on Information Technology, Computer, and Electrical Engineering (ICITACEE).

[27] Li T.-H.S., Chiu H.-J., Kuo P.-H. (2022). Hepatitis C Virus Detection Model by Using Random Forest, Logistic-Regression and ABC Algorithm. IEEE Access.

[28] Terlapu P.V., Gedela S.B., Gangu V.K., Pemula R. (2022). Intelligent Diagnosis System of Hepatitis C Virus: A Probabilistic Neural Network Based Approach. Int. J. Imaging Syst. Technol..

[29] Kaunang F.J. (2022). A Comparative Study on Hepatitis C Predictions Using Machine Learning Algorithms. 8ISC Proceedings: Technology, [S.l.]. http://ejournal.unklab.ac.id/index.php/8ISCTE/article/view/684.

[30] Safdari R., Deghatipour A., Gholamzadeh M., Maghooli K. (2022). Applying Data Mining Techniques to Classify Patients with Suspected Hepatitis C Virus Infection. Intell. Med..

[31] Ferdib-Al-Islam, Akter L., Khanna A., Gupta D., Bhattacharyya S., Hassanien A.E., Anand S., Jaiswal A. (2022). Detection of Hepatitis C Virus Progressed Patient’s Liver Condition Using Machine Learning BT—International Conference on Innovative Computing and Communications.

[32] Kim I.H., Kisseleva T., Brenner D.A. (2016). Aging and Liver Disease. https://pubmed.ncbi.nlm.nih.gov/25850346/.

