# Supplementary material for: Performance Comparison of Machine Learning Approaches on Hepatitis C Prediction Employing Data Mining Techniques
Source: Bioengineering (Basel). 2023 Apr 17;10(4):481. doi: 10.3390/bioengineering10040481 (PMC10135598; doi:10.3390/bioengineering10040481)
Supplement: Supplementary file 1 [file bioengineering-10-00481-s001.zip › bioengineering-2324497-supplementary.pdf]

Supplementary materials:

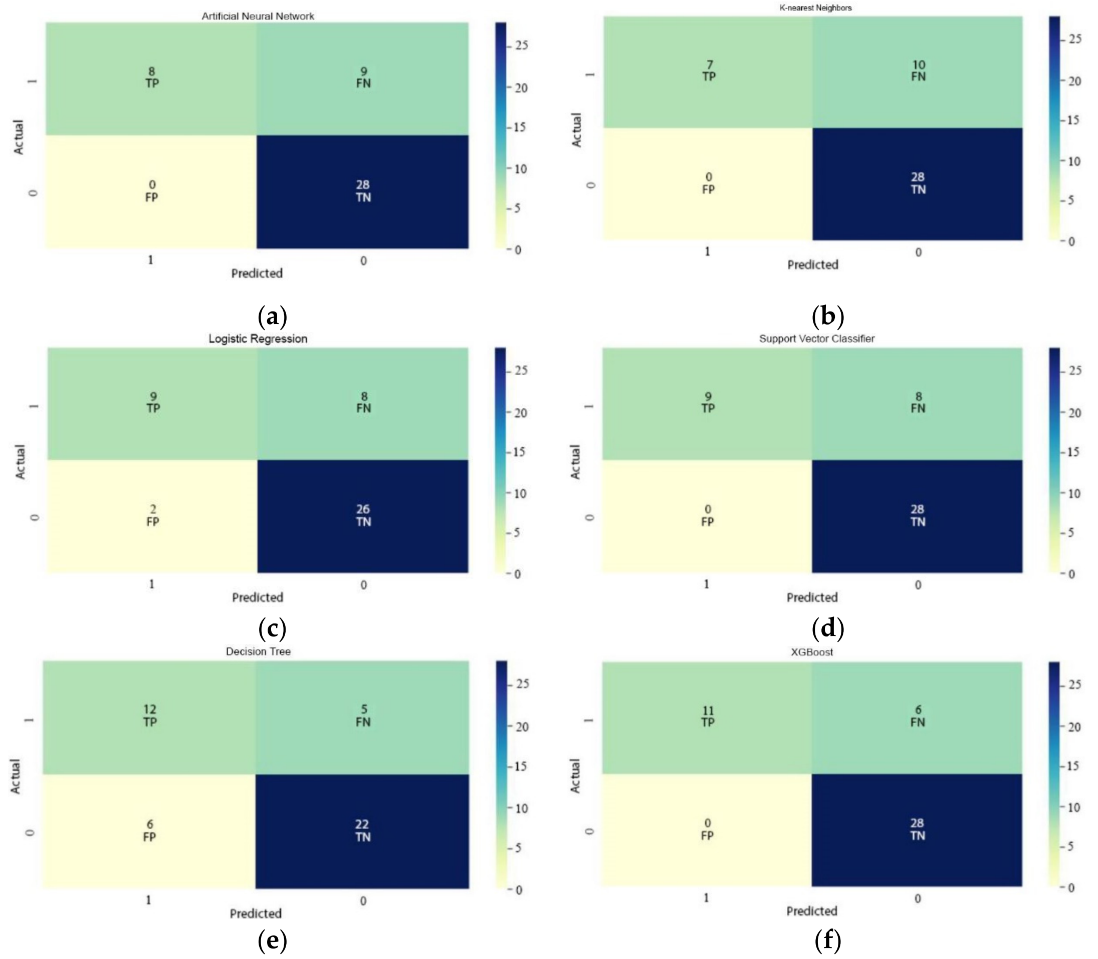

Figure S1. Confusion matrix of six machine learning algorithms, (a) Artificial Neural Network (b) k-nearest Neighbors (c) Logistic Regression (d) Support Vector Machines (e) Decision Tree (f) XGBoost, with the NHANES Dataset.

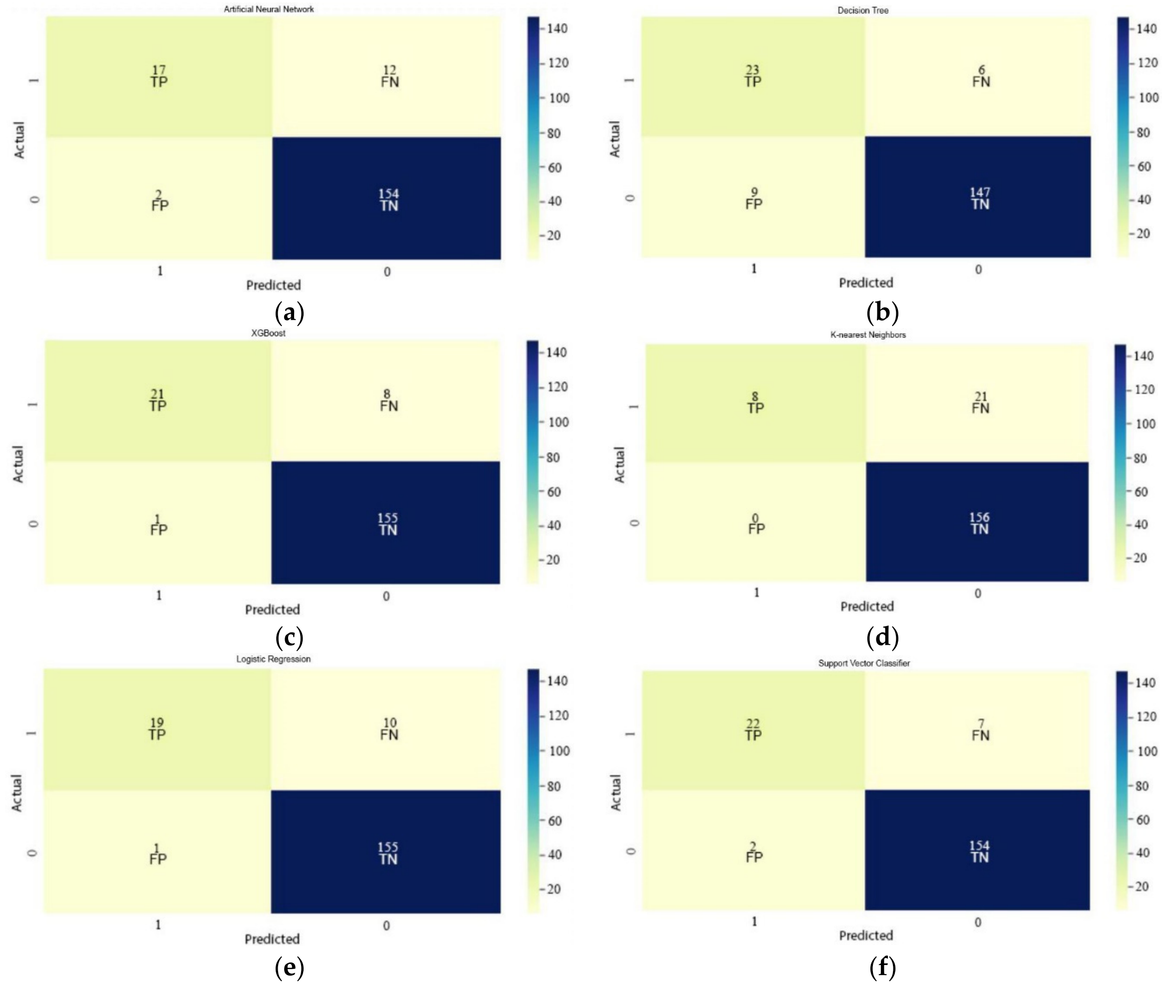

Figure S2. Confusion matrix of six machine learning algorithms, (a) Artificial Neural Network (b) Decision Tree (c) XGBoost (d) k-nearest Neighbors (e) Logistic Regression (f) Support Vector Machines, with the UCI dataset.
